# A mixed presentation of IgA nephropathy and malignant hypertension with histological thrombotic Microangiopathy

**DOI:** 10.1093/omcr/omag047

**Published:** 2026-04-28

**Authors:** Michael Isaacs, Uzzal K Talukdar, Krishna Gokula, Clyson Mutatiri

**Affiliations:** Department of Medicine, Bundaberg Hospital, Bundaberg 4670, Australia; Faculty of Medicine, University of Queensland, Brisbane 4006, Australia; Department of Medicine, Bundaberg Hospital, Bundaberg 4670, Australia; Department of Medicine, Bundaberg Hospital, Bundaberg 4670, Australia; Renal Medicine, Bundaberg Hospital, Bundaberg 4670, Australia; Bundaberg Clinical School, University of Queensland, Bundaberg 4670, Australia

**Keywords:** nephrology, IgA nephropathy, thrombotic Microangiopathy, hypertension, malignant hypertension, acute kidney injury, chronic kidney disease

## Abstract

IgA Nephropathy (IgAN) is one of the most common causes of glomerulonephritis. Biopsy is necessary for definitive diagnosis, though due to variability in severity and progression patients may forego biopsy initially. However, certain clinical presentations and histopathological findings may be significant predictors of poor prognosis. We describe a case of IgAN initially presenting as malignant hypertension. Retrospective chart review suggested previous hospital encounters with features of IgAN, before loss to follow up. Subsequent biopsy evidence of co-occurrent IgA deposition and thrombotic microangiopathy were identified. The occurrence of severe hypertension and the aforementioned biopsy findings in IgAN are rare, and strong predictors of rapid progression to end-stage kidney disease. We briefly review the literature supporting this prognosis.

## Introduction

IgA nephropathy (IgAN) is one of the most common causes of glomerulonephritis [1, 2]. It is often asymptomatic, though clinically significant disease can range from benign to rapidly progressive. Benign presentations of suspected IgAN may be managed conservatively in the absence of reduced renal function [1]. This wide variation makes its true prevalence uncertain.

Severe IgAN is a difficult clinical entity to predict. Other than rapidly progressive glomerulonephritis, there are certain uncommon clinical features that predict rapid progression [1–3]. We describe a case of IgAN presenting as hypertensive crisis with histological thrombotic microangiopathy (TMA). These features are significant both for their uncommon occurrence and overlapping underlying pathophysiology. We review the literature regarding hypertension and histological TMA in IgAN as predictors of poor renal prognosis.

## Case report

A 28 year old male was referred to our hospital after outpatient optometry appointment detected bilateral papilloedema following 2 weeks of headaches, blurred vision, and associated nausea and vomiting. His background was significant for schizophrenia, stable on olanzapine and valproate. Two previous episodes of haematuria at 18 and 22 years of age were noted: the first episode was associated with a transient acute kidney injury (AKI). He had attended outpatient renal clinic for two years—renal scintigraphy scan and blood pressure (BP) measurements at that time were normal, and a presumptive diagnosis of IgAN was determined. He opted for conservative management; steroids or RAAS agents were not prescribed. The patient declined renal biopsy and was subsequently lost to follow up.

On this admission, he was noted to have a BP of 211/132 mmHg. Initial blood work demonstrated a significant AKI (Urea 22.9 mmol/l, Creatinine 403 μmol/l, estimated Glomerular Filtration Rate (eGFR) 16 ml/min/1.73m^2^; 2.5 years prior Urea 5.6, Creatinine 90, eGFR > 90). Urine output was maintained. Examination demonstrated no stigmata of renal or hypertensive disease. He was admitted to the Intensive Care Unit (ICU) for 3 days for treatment of hypertensive emergency. He was managed initially with a labetalol infusion and continuous IV saline, transitioning to oral labetalol and prazosin on stepdown to the medical ward. Amlodipine and isosorbide mononitrate were added to maintain systolic BP < 150 mmHg. He experienced an episode of peripheral oedema, resolving with 5% albumin administration.

**Figure 1:**
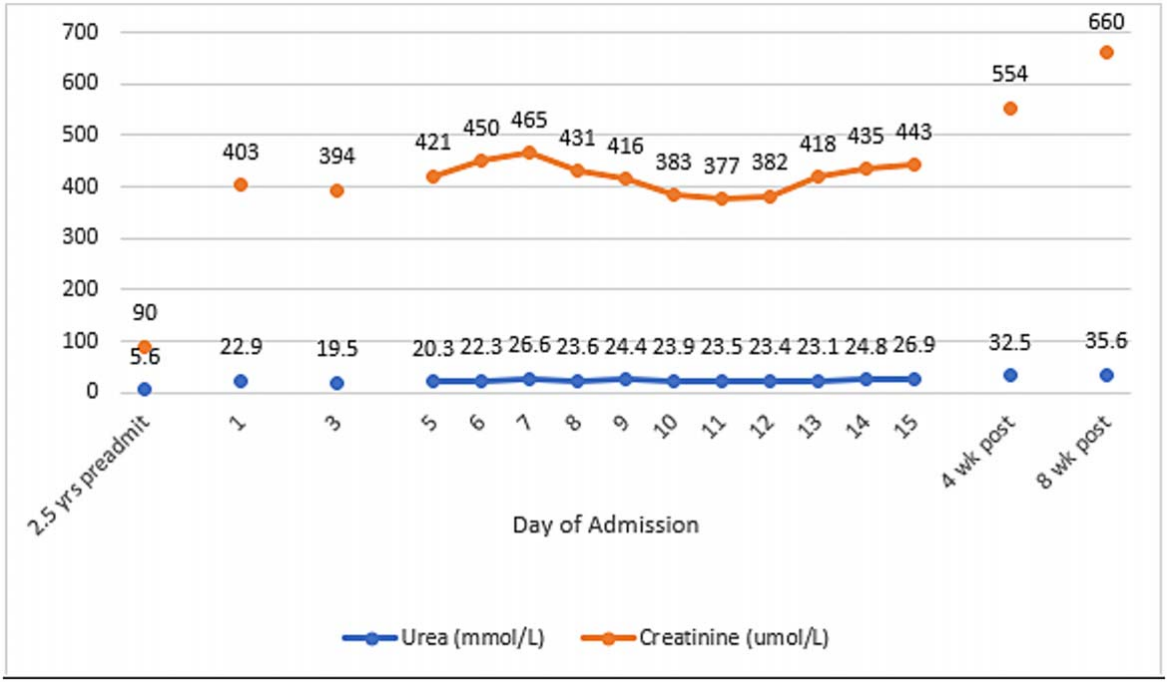
Graph of renal function biochemistry over time, including previous baseline 2.5 years prior to admission. 2.5 years pre-admission eGFR = > 90 mL/min/1.73 m2, pre-discharge eGFR = 15, 8 week follow-up eGFR = 9.

**Figure 2:**
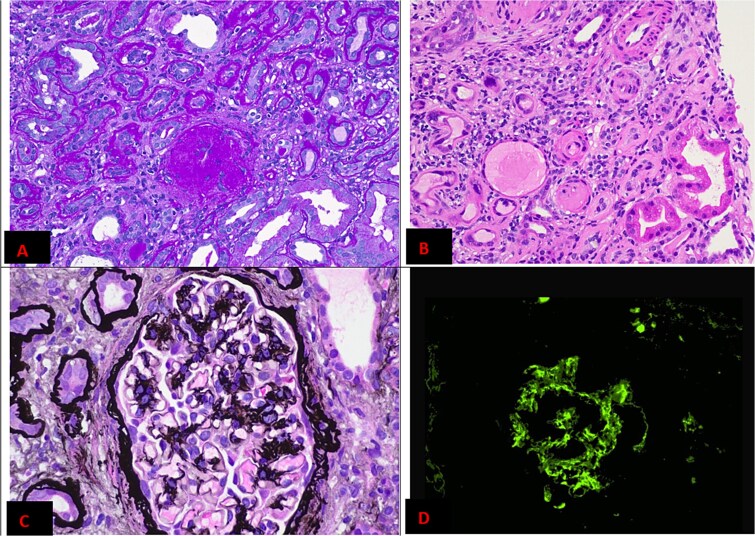
Histopathology slides from the renal biopsy demonstrating: (A) global glomerular sclerosis; (B) abnormal arterioles; (C) mesangial proliferation; and (D) IgA deposition on immunofluorescence. See appendix 1 for full report.

Despite treatment, his AKI did not improve (Fig. 1). Further investigation was undertaken to determine cause (Table 1). Ultrasonography of his urinary tract was undertaken, demonstrating increased parenchymal echogenicity with intact corticomedullary differentiation, consistent with early renal parenchymal disease. Urine microscopy showed erythrocytes > 500 × 10^6^/l and urine protein-to-creatinine ratio (PCR) from an average of 3 collections was 397 g/mol, elevated compared to 10 years prior (PCR 81). On day 7 of his admission a renal biopsy was undertaken (Fig. 2). Extensive global glomerular sclerosis was noted, with moderate mesangial expansion and pseudocrescents. Arterial hyalinosis and subendothelial narrowing was observed, and one artery demonstrated thrombosis. Extensive tubular atrophy and interstitial fibrosis was also noted. Immunofluorescence demonstrated IgA deposition.

**Table 1:** Screening tests ordered to determine cause of renal injury.

Test	Result (Ref. Range)	Test	Result (Ref. Range)
Urine M/C/S	Leukocytes < 10 (< 10) **Erythrocytes > 500** (< 10) Epithelials < 10 (< 10) **Hyaline Casts 3**	Metabolic Screen – Hba1c – Triglycerides – LDL – HDL	5.1% (4.3–6.0) 1.2 mmol/l (< 1.5) 2.3 mmol/l (< 3.0) 1.0 mmol/l (> 1.0)
Urine Protein/Electrolytes – Sodium – Potassium – Chloride – Creatinine – Protein – Albumin – P/Cr – A/Cr	84 mmol/l 21 mmol/l 77 mmol/l 4.6 mmol/l **1400 mg/l** (< 100) **1300 mg/l** (< 20) **345 g/mol** (< 15) **340 g/mol** (< 1.0)	Cortisol (random) Aldosterone Renin Aldosterone/Renin Ratio Metadrenalines – Normetadrenaline – Metadrenaline – 3-Methoxytyramine	223 nmol/l (140–640) 257 pmol/l (0–400) 10.1 mU/l (2–29) 26 (< 55) 598 pmol/l (120–600) 208 pmol/l (30–450) 25 pmol/l (< 90)
Urine drugs of abuse – Benzodiazepines – Canabinoids – Amphetamines – Opioids	Negative Negative Negative Negative	Haemolysis/TTP – Direct Coombs – Haptoglobin – ADAMTS-13 – Reticulocytes	**Positive (C3d)** 0.51 g/l (0.40–2.80) > 0.82 U (0.61–1.31) 38 × 10^9/l (10–100)
24 hr Urine – Volume – Creatinine – HMMA – Normetadrenaline – Metadrenaline – 3-Methoxytyramine	4 l **18.4 mmol** (8.0–18.0) **27 umol** (< 25) **2.4 umol** (< 2.3) 1.2 umol (< 1.7) 0.7 umol (< 1.3)	Viral Screening – Quantitative HCV – HCV Ab IgG – HBVsAg – HBVcAb – HIV – CMV IgG – CMV IgM	Not Detected Non-Reactive Non-Reactive Non-Reactive Non-Reactive **Reactive** Non-Reactive
Full Blood Count – Haemoglobin – MCV – White Cell Count – Neutrophils – Platelets Biochemistry – Sodium – Chloride – Potassium – Magnesium – Calcium (Corrected) – Phosphate – LDH Venous Blood Gas – pH – Bicarbonate – pCO2	**129 g/L** (135–180) 80 fL (80–100) **12.6 × 10^9/L** (4.0–11.0) **10.81 × 10^9/L** (2.00–8.00) 269 × 10^9/L (140–400) 140 mmol/l (135–145) 109 mmol/l (95–110) 5.1 mmol/l (3.5–5.2) 0.86 mmol/l (0.70–1.10) 2.50 mmol/l (2.10–2.60) 1.49 mmol/l (0.75–1.50) 246 U/L (120–250) 7.32 (7.32–7.43) **20 mmol/l** (22–33) 40 mmHg (38–54)	Autoimmune Screen – ANA – ENA – Rheumatoid Factor – Anti-CCP – Anti-TTG – c-ANCA – p-ANCA – C3 – C4 – IgA	Negative Negative < 20 U (< 20) Negative < 2 U (< 2) Negative Negative 1.31 g/l (0.90–1.80) 0.27 g/l (0.10–0.40) 3.4 g/l (1.0–4.0)

Bold indicates abnormal value.

Two separate histological findings were apparent: IgAN and TMA. He was discharged on day 15 after his renal function stabilised. Follow-up in outpatient clinic demonstrated progressive deterioration of his kidney function at 4 and 8 weeks post-discharge. BP control had improved but remained suboptimal; irbesartan, calcium, and bicarbonate supplementation were introduced. He was referred for vascular access preparation and transplant workup at last outpatient review.

## Discussion

In IgAN, deposition of IgA in the glomerular mesangium leads to impaired podocyte function and mesangial proliferation [1, 2]. The classic presentation is a nephritic picture: episodic haematuria may be evident, often following upper respiratory tract infection. Clinical factors and renal dysfunction may allow for presumptive diagnosis; biopsy is necessary for definitive diagnosis. However, in benign presentations biopsy may not always be performed immediately—as in the case of our patient [1]. More severe presentations can present as rapidly progressive glomerulonephritis resulting in kidney failure—in these cases, crescents will generally be noted on histopathology. This contrasts with our patient, whose histopathological findings were mixed.

In studies of patients with IgAN, the incidence of TMA appears to be relatively uncommon. Most identify incidence of around 2%–15%, though one study demonstrated 53% incidence [4–8]. It is postulated that the pro-inflammatory state of IgAN leads to complement and coagulation cascade activation, with resultant endothelial damage and thrombus formation [5]. Histopathology of these patients demonstrates higher intensity complement deposition [8]. Our patient’s histopathology demonstrated 3+ C3 mesangial reactivity, which is consistent with this literature. Histopathologic TMA can be an independent marker of disease severity: progression to end stage kidney disease is up to twice as rapid despite best management in these patients [3, 9].

The classic presentation of primary TMA demonstrates haematuria and hypertension—symptoms common to IgAN [8]. Secondary TMA can also manifest as a complication of hypertensive disease—likely secondary to direct pressure-related endothelial damage [4]. While hypertension is commonly seen in IgAN [1], malignant hypertension is certainly less common [3]. By itself, hypertension is a strong predictor for IgAN progression [3]. It is clear from the cohort studies that TMA in the context of IgAN is strongly associated with malignant or uncontrolled hypertension. However, it is unclear whether its manifestation is secondary to the hypertension or the proinflammatory state of IgAN.

Regardless, the co-occurrence of TMA and hypertension is a strong predictor of progression of disease—up to 70% of patients progress to end stage kidney disease within a year [3]. For our patient, biopsy findings demonstrated Oxford grading M0 E0 S1 T1 C0; the International IgAN Prediction Tool [10] calculated risk of 50% decline in eGFR or progression to end-stage kidney disease was only 33.92% at 2 years. Despite these predictive indices and improved BP control, our patient’s kidney function continued to decline. The further deterioration in kidney function is only partially explained by introduction of irbesartan.

Our case demonstrates the importance of this presentation in predicting the outcome of IgAN. Hypertensive changes and TMA on histopathology have overlapping pathophysiology, but appear to independently predict progression of disease. Awareness of these factors is important to adequately weigh risk of progression in otherwise seemingly benign presentations. Consideration should be given for incorporation of TMA into IgAN risk calculation tools.

## Supplementary Material

Appendix_1_omag047
