# Supplementary material for: A mixed presentation of IgA nephropathy and malignant hypertension with histological thrombotic Microangiopathy
Source: Oxf Med Case Reports. 2026 Apr 28;2026(4):omag047. doi: 10.1093/omcr/omag047 (PMC13122600; doi:10.1093/omcr/omag047)
Supplement: Appendix_1_omag047 [file appendix_1_omag047.docx]

**Appendix 1:** Full histopathology report of renal biopsy

*Glomeruli: There are up to 11 glomeruli per slide; 8 (73%) are globally sclerosed. The non-sclerosed glomeruli show moderate expansion of mesangial regions without significant mesangial hypercellularity. Two remaining glomeruli show prominent cells in Bowman's space, which in my opinion represent podocytic hyperplasia (pseudocrescents) rather than true crescents. Some cells contain protein resorption droplets, and one glomerulus contains a space between the parietal epithelium and the enlarged cells; they also lack fibrin and neutrophils. One glomerulus shows segmental sclerosis (slide 7).*

*Vessels: One artery is included, and it appears normal. There is focal arteriolar hyalinosis. Several other arterioles show abnormal features. One contains a thrombus that extends into the hilum of a sclerosed glomerulus (slide 16). Others show subendothelial swelling and narrowing of the lumen. There is no vascular inflammation.*

*Tubules and interstitium: There is tubular atrophy and interstitial fibrosis involving approximately 50% of the cortex. There appears to be a degree of compensatory tubular epithelial hyperplasia. Other non-atrophic tubules show features of acute injury including cytoplasmic vacuolation, reactive nuclear changes, and mitotic activity. The areas of atrophy in the biopsy contain an infiltrate of lymphocytes with rare plasma cells and eosinophils.*

*Immunofluorescence: The tissue submitted for IF comprises renal tissue with 5 non-sclerosed glomeruli and one sclerosed glomerulus per slide. Reactivity is as follows (0-3+ scale):*

*IgG, IgM, C1q: Negative*

*IgA: Mesangial (3+)*

*C3: Mesangial in glomerular capillary wall (2+)*

*Kappa light chain: Trace mesangial (+/-)*

*Lambda light chain: Mesangial (2+)*

*Electron microscopy: Not performed.*

***SUMMARY***

*Primary glomerular diagnosis: IgA nephropathy*

*Pattern of glomerular injury: Mild mesangial changes with no true crescents*

*Oxford grade: M0 E0 S1 T1 C0*

*Additional features: Global glomerulosclerosis (73%), moderate (50%) tubular atrophy and interstitial fibrosis, no arteriosclerosis*

*Secondary diagnosis: Arteriolar changes consistent with thrombotic microangiopathy; acute tubular injury*

*Chronicity grade: Moderate (total renal chronicity score 5/10)*

***COMMENT***

*This biopsy appears to contain two pathological processes. Firstly, there is IgA nephropathy characterised by strong IgA reactivity and mesangial matrix expansion in the non-sclerosed glomeruli. Secondly, there are changes in the arterioles which are highly suspicious for thrombotic microangiopathy. There is extensive global glomerulosclerosis in association with moderate to severe atrophy*
